# Heat shock factor binding in Alu repeats expands its involvement in stress through an antisense mechanism

**DOI:** 10.1186/gb-2011-12-11-r117

**Published:** 2011-11-23

**Authors:** Rajesh Pandey, Amit K Mandal, Vineet Jha, Mitali Mukerji

**Affiliations:** 1 Genomics and Molecular Medicine, Institute of Genomics and Integrative Biology, Council of Scientific and Industrial Research (CSIR-IGIB), Mall Road, Delhi-110007, India; 2 GN Ramachandran Knowledge Centre for Genome Informatics, Institute of Genomics and Integrative Biology, Council of Scientific and Industrial Research (CSIR-IGIB), Mall Road, Delhi-110007, India

## Abstract

**Background:**

Alu RNAs are present at elevated levels in stress conditions and, consequently, Alu repeats are increasingly being associated with the physiological stress response. Alu repeats are known to harbor transcription factor binding sites that modulate RNA pol II transcription and Alu RNAs act as transcriptional co-repressors through pol II binding in the promoter regions of heat shock responsive genes. An observation of a putative heat shock factor (HSF) binding site in Alu led us to explore whether, through HSF binding, these elements could further contribute to the heat shock response repertoire.

**Results:**

Alu density was significantly enriched in transcripts that are down-regulated following heat shock recovery in HeLa cells. ChIP analysis confirmed HSF binding to a consensus motif exhibiting positional conservation across various Alu subfamilies, and reporter constructs demonstrated a sequence-specific two-fold induction of these sites in response to heat shock. These motifs were over-represented in the genic regions of down-regulated transcripts in antisense oriented Alus. Affymetrix Exon arrays detected antisense signals in a significant fraction of the down-regulated transcripts, 50% of which harbored HSF sites within 5 kb. siRNA knockdown of the selected antisense transcripts led to the over-expression, following heat shock, of their corresponding down-regulated transcripts. The antisense transcripts were significantly enriched in processes related to RNA pol III transcription and the TFIIIC complex.

**Conclusions:**

We demonstrate a non-random presence of Alu repeats harboring HSF sites in heat shock responsive transcripts. This presence underlies an antisense-mediated mechanism that represents a novel component of Alu and HSF involvement in the heat shock response.

## Background

Alu repeats, which occupy more than one-tenth of the human genome, have been shown to harbor a large number of transcription factor binding sites (TFBSs) [1-3], many of which have also been demonstrated to be functionally active. These have been mostly discovered during the course of characterization of regulatory sites in promoter regions of genes [4-15]. Recently, genome wide informatics analyses have revealed substantial distribution of these sites in Alu repeats - for instance, nearly 90% of retinoic acid response element binding sites in human are in Alus [16]. As Alus also provide substrates for non-homologous recombination, they are also enriched in a large number of regions of segmental duplication [17,18]. Through these recombination events, Alus harboring regulatory sites could also create novel regulatory networks. We have shown earlier that not only are Alus non-randomly distributed but they also selectively retain regulatory sites in genes of specific biological processes [19,20]. This reiterates that these elements are not passive members of the genome [21-23]. So far, however, genome-wide effects of these elements have not been demonstrated.

Alu elements not only provide accessory sites for transcription factor binding together with RNA polymerase (pol) II [1] but can themselves be transcribed by RNA pol III [24,25]. Alu RNA levels have been shown to be elevated in heat shock stress [26] and these RNAs are reported to act as transcriptional co-repressors through binding to RNA pol II in response to heat shock [27]. A preliminary analysis through an in-house developed tool has revealed the presence of heat shock factor (HSF) binding sites in Alu elements. Since Alu elements have been widely implicated in stress responses [28-30], we decided to investigate if HSF, through binding to these sites in Alus, could also modulate genome-wide expression in response to heat shock.

Our results demonstrate that HSF sites in Alu elements are significantly over-represented in heat shock responsive transcripts and these sites also bind HSFs and are responsive to heat shock in minimal promoter constructs. Besides, the density of Alu elements harboring HSF sites are over-represented in the genic regions of down-regulated transcripts compared to up-regulated ones and many of these down-regulated transcripts also have antisense transcripts. This study not only adds a novel dimension of Alu involvement in heat shock response but also highlights the potential of these elements in the evolution of novel regulatory networks in primate lineages.

## Results

### Significant enrichment of Alu repeats in heat shock responsive transcripts

Genome-wide expression analysis in response to heat shock using the Illumina BeadChip revealed 1,284 up-regulated and 2,995 down-regulated transcripts (Additional file 1). Comparison of Alu density in the upstream 5 kb and downstream genic regions with a random set of genes revealed significant enrichment of Alus in the proximity of the differentially expressed transcripts (Table 1). Interestingly, both in the upstream as well as downstream regions, the enrichment was significantly higher in the down-regulated transcripts, with more conspicuous differences observed in the genic regions.

**Table 1:** Comparison of Alu density between heat shock responsive transcripts in upstream and genic regions and randomly selected genes

	Comparison of Alu density	Regions	
		
S.No	(two-tailed *t*-test)	*P*-value (upstream)	*P*-value (genic)
1	Up-regulated versus random	0.036	Not significant
2	Down-regulated versus random	0.020	7.48 × 10^-18^
3	Down-regulated versus up-regulated	0.027	4.46 × 10^-22^

Genes were randomly selected from UCSC RefSeq genes (hg18). S.No: Serial number

### HSF sites in Alus are positionally conserved and heat shock responsive

**Figure 1:**
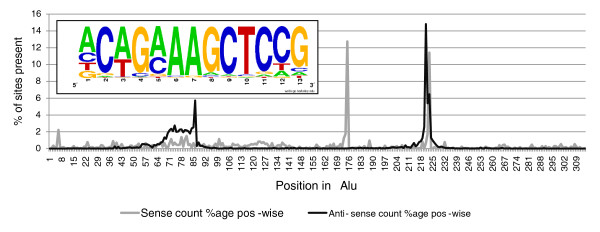
**Heat shock factor binding motif and preferred binding sites in Alu repeats of heat shock responsive transcripts**. The frequency of three preferred binding sites of HSFs mapped on to Alu repeats, in both sense and antisense orientations, of heat shock responsive transcripts is shown. The HSF site at position 221 is common between both sense and antisense Alus. Sites at positions 175 and 91 are unique to sense and antisense Alus, respectively. The inset shows the consensus motif for HSF sites across all Alu subfamilies.

**Figure 2:**
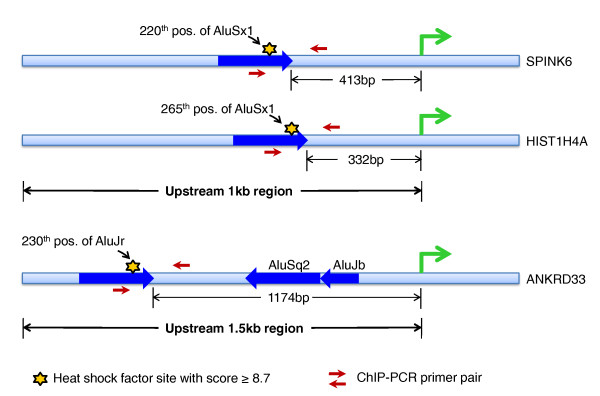
**Location of validated heat shock factor sites within Alus in the promoter region of up-regulated genes**. A substantial fraction of up-regulated genes harbor high score (≥8.7) HSF sites within Alus in the upstream region. All the validated genes (*SPINK6*, *HIST1H4A *and *ANKRD33*) have their first HSF site within Alus in the promoter region from the transcription start site. Functional validation of such HSF sites through cloning, site-directed mutagenesis, transient transfection in a cell line and chromatin immunoprecipitation (ChIP) followed by PCR confirmed their role in the heat shock response.

**Figure 3:**
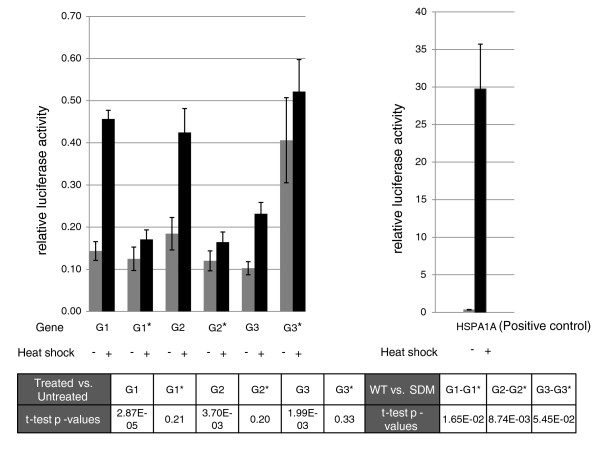
**Heat shock factor sites in Alu repeats are functionally active**. Reporter constructs of the promoter region of three genes, *SPN *(G1), *SPINK6 *(G2) and *HIST1H4A *(G3), containing a HSF site within Alus when cloned downstream of a minimal promoter containing a firefly luciferase construct show more than two-fold induction in response to heat shock. Site-directed mutagenesis (SDM) in the predicted HSF within Alus led to reduced induction of approximately 1.4-fold in response to heat shock stress. Data were normalized using co-transfected renilla luciferase vector. The experiment was repeated three times and in triplicates to analyze standard deviations in all cases. *HSPA1A *was used as a positive control after an earlier published report of the functional HSF in the promoter region of the gene; the HSF site is in a non-Alu region of the *HSPA1A *gene promoter. G1, G2 and G3 represent wild type (WT) constructs, whereas G1*, G2* and G3* represent the SDM constructs. The statistical significance (Student's *t*-test) of the observed expression changes in response to heat shock in WT and SDM constructs as well as between them are shown in the lower panel, where figures in bold represent a significant *P*-value change. It is worth mentioning that the SDM constructs do not show significant expression changes following heat shock whereas WT and WT versus SDM show significant changes in expression. Error bars represent standard deviation among experimental replicates.

**Figure 4:**
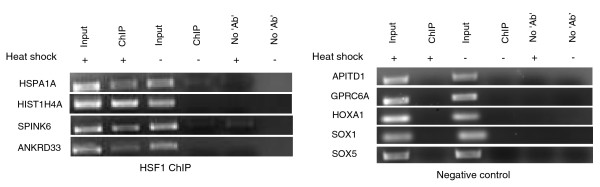
**Heat shock factor binds to its cognate site in Alu repeats**. The binding of a HSF to the heat shock element sequence present within the Alu repeat of *HIST1H4A*, *SPINK6 *and *ANKRD33 *genes in the promoter region was confirmed by ChIP-PCR after heat shock treatment. *HSPA1A*, with a HSF binding site in a non-Alu region, was used as a positive control. Input chromatin was used to ascertain that equal amounts of chromatin were applied in each reaction. Enrichment in heat shock treated samples compared to untreated and no antibody (Ab) ChIP samples is clearly visible. The faint band in the untreated *HSPA1A *sample confirms earlier reports of binding of HSF to this promoter, although this is not induced. As negative controls we considered regions of the genome that do not contain an HSF site. The negative controls do not show any band in the treated/untreated ChIP or 'no Ab' lanes, although the band in the input chromatin lane confirms the genomic presence of the queried region.

The previously determined consensus human heat shock element bound by HSF is nTTCnnGAAnnTTCn [31]. In our study, we predict a novel consensus HSF motif of 13 bp, nCAGAAAGCTCCG (Figure 1), harbored within Alu repeats. These binding sites within Alus show positional conservation and are present across different subfamilies of Alus in genic or upstream regions of all the differentially expressed transcripts (Additional file 2). We observed two preferred HSF binding sites in sense and antisense Alus. The HSF site at position 221 overlaps in both orientations whereas at positions 175 and 91 they are uniquely present in sense and antisense Alus, respectively. We selected a representative set of high score (≥8.7) putative HSF sites within Alus present in the promoter region of up-regulated genes for functional validation. A schematic showing the location of validated HSF sites within Alus is provided in Figure 2. It is noteworthy to mention that the first HSF site in these genes was within the Alu repeats from the transcription start site and there was no intervening HSF present in non-Alu regions. These HSF sites within Alus were observed to confer more than two-fold induction of the luciferase reporter gene in response to heat shock (Figure 3). The induction was reduced to approximately 1.4-fold when specific mutations were made in the most conserved consensus HSF motif within Alus (Figure 3). Although the induction was significantly lower compared to that of the positive control gene *HSPA1A*, the more than two-fold induction was consistent across Alus from the three genes that were studied. Moreover, the fold changes observed in wild-type and mutated HSF sites in response to heat shock were statistically significant (Figure 3). The binding of HSFs to the predicted heat shock element within Alus was confirmed by chromatin immunoprecipitation (ChIP)-PCR. We used *HSPA1A*, which harbors an HSF site in a non-Alu region, as positive control (Figure 4). In order to determine the specificity of HSF binding to its cognate site within Alus, we also included genomic regions that do not contain an HSF site as negative controls.

### Contrasting patterns of accumulation of HSF sites in Alu repeats in upstream and genic regions

A search for HSF motifs in the upstream 5-kb and downstream genic regions of heat shock responsive transcripts revealed their presence in a substantial fraction of Alu repeats. We next compared the relative abundance of these sites in Alu versus non-Alu regions, in both the upstream and genic regions of differentially expressed transcripts (Additional file 3).

**Figure 5:**
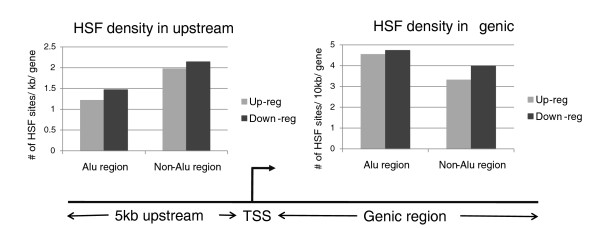
**Contrasting patterns of HSF distribution in Alu and non-Alu sequences in upstream and genic regions of heat-shock responsive transcripts**. In upstream regions, the non-Alu region had significantly higher HSF site density (*P*-value 2.05 × 10^-24 ^and 1.8 × 10^-20 ^for up-regulated and down-regulated transcripts, respectively). In genic regions, Alu regions have significantly higher HSF sites than non-Alu regions for both up-regulated (*P*-value 8.97 × 10^-13) ^and down-regulated (*P*-value 2.58 × 10^-3^) transcripts.

It is evident from Table 2 that HSF sites are present both within and outside Alus in nearly 50% of the differentially expressed transcripts in both genic and upstream regions. As is evident from the table, there is a set of transcripts with HSF sites exclusively in 'Alu' or 'non-Alu' regions. Further, we observe significant differences in HSF density between Alu and non-Alu regions (Additional file 4). Interestingly, these differences are in opposite directions in the upstream and genic regions (Figure 5). Whereas in upstream the non-Alu regions had significantly higher HSF site density (*P*-value 2.05 × 10^-24 ^and 1.8 × 10^-20 ^for up-regulated and down-regulated transcripts, respectively, the reverse was observed for genic regions. In the latter, Alu regions have significantly higher HSF sites than non-Alu regions for both up-regulated (*P*-value 8.97 × 10^-13^) and down-regulated (*P*-value 2.58 × 10^-3^) transcripts.

**Table 2:** HSF site distribution in Alu and non-Alu regions in heat shock responsive transcripts across the upstream 5-kb and genic regions

Region	HSF sites	Up-regulated	Down-regulated
Upstream	Alu + non-Alu	731	1,592
	Exclusively in Alu	60	184
	Exclusively in non-Alu	430	833
Genic	Alu + non-Alu	775	1,737
	Exclusively in Alu	12	13
	Exclusively in non-Alu	238	433

### Alu repeats in antisense orientation have more HSF sites

We observed a biased distribution of Alu repeats harboring HSF sites according to their orientation, sense or antisense, with respect to the host transcript (Additional file 3). As is evident from Table 3, though there is no comparable difference in the number of Alu repeats in sense and antisense orientations, HSF sites in antisense Alus are significantly (four- to six-fold) over-represented; in contrast to approximately 4% of sense Alus harboring a HSF site, nearly 21% of antisense Alus harbor one. Most noteworthy was the high number of HSF sites within antisense Alus in the genic region of down-regulated transcripts. This led us to hypothesize that down-regulation in response to heat shock could be mediated through antisense transcripts driven by HSF binding in the antisense Alus.

**Table 3:** Alu and HSF site distribution in sense and antisense orientation in heat shock responsive transcripts in the upstream 5-kb and genic regions

Expression	Regions	Sense Alu	Antisense Alu	HSF sites in sense Alu	HSF sites in antisense Alu
Up-regulated	Upstream	2,301	2,393	112	481
	Genic	14,580	17,507	632	3,629
Down-regulated	Upstream	6,244	7,187	268	1,512
	Genic	47,295	63,323	1,961	13,092

HSF sites are significantly enriched within antisense Alus present in the genic region of down-regulated transcripts. Although HSF sites within antisense Alus are enriched across all categories, the biased distribution is more evident in the genic region of down-regulated transcripts.

### HSF sites in down-regulated transcripts could drive antisense transcripts

**Figure 6:**
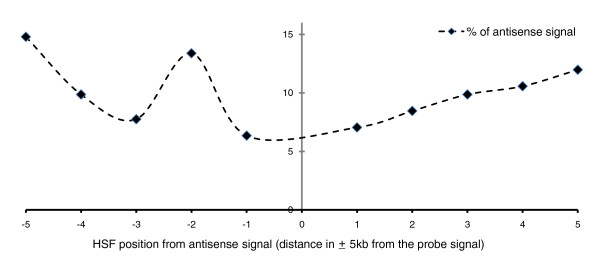
**Presence of Alus containing HSF sites in 5-kb proximal regions of antisense signals**. HSF sites are especially enriched in the upstream (approximately 2-kb) region of the antisense transcript signal. Antisense transcripts may be transcribed through HSF binding to Alu repeats, leading to down-regulation of sense transcripts in response to heat shock.

**Figure 7:**
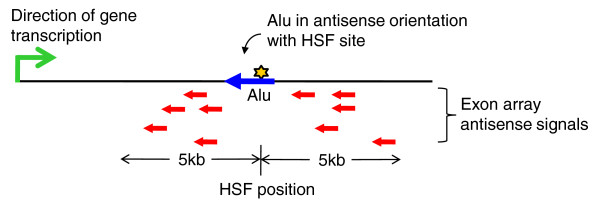
**Schematic for exon array antisense signals observed in heat shock down-regulated genes**. Exon array probe co-ordinates for antisense signals are within 5-kb of HSF sites in antisense-oriented Alus. This led us to hypothesize that such Alus harboring high score (≥8.7) HSF sites can initiate antisense transcripts, leading to gene down-regulation through either transcriptional interference or the sense-antisense-mediated RNA interference pathway.

In order to test the above hypothesis, we carried out expression analysis of antisense transcripts in response to heat shock using a modified Affymetrix exon array protocol. A common set of 381 transcripts were down-regulated after heat shock with both Illumina microarray and affymetrix Exon array analysis. Of these transcripts, 268 harbored HSF sites within Alus in the genic region. Amongst these, we observed signals in 136 transcripts in the antisense array (Additional file 5). In order to check whether the presence of HSF sites in Alus could drive antisense transcription, we looked at the relative proximity of the HSF sites in Alus with respect to antisense transcripts. When we applied a threshold criteria of a 5-kb flanking region to the antisense transcript, we observed HSF sites in 78 genes (Figure 6; Additional file 5). Of these, most (51 genes) are in the upstream region, with enrichment around the 2-kb region of the antisense signal. A schematic representation of the exon array probes for expression of antisense transcripts following heat shock stress is shown in Figure 7. Functional annotation clustering (using DAVID) of the antisense signals revealed significant enrichment of processes related to transcription from the RNA pol III promoter and these genes were a part of the transcription factor IIIC complex (TFIIIC) complex in the nucleoplasm (Table 4).

**Table 4:** Functional annotation clustering of down-regulated transcripts with antisense transcript signal

Functional category	Functional class	*P*-value (multiple testing)/FDR
Biological process	GO:0006383: transcription from RNA polymerase III promoter	0.42/0.85
	GO:0032869: cellular response to insulin stimulus	0.50/4.25
	GO:0051276: chromosome organization	0.45/4.61
Cellular component	GO:0044451: nucleoplasm part	9.1 × 10^-5^/5.1 × 10^-4^
	GO:0000127: transcription factor TFIIIC complex	0.02/1.2
	GO:0000123: histone acetyltransferase complex	0.12/9.26
Annotation cluster	IPR019787: zinc finger, PHD-finger	0.01/0.06

### Antisense transcripts sequester sense transcripts during heat shock stress

**Figure 8:**
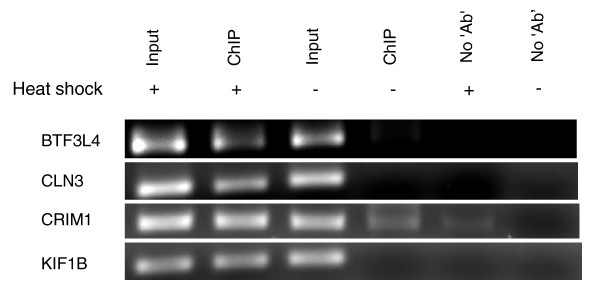
**HSF occupancy within Alus in the proximal region of antisense transcripts**. The possibility that putative high-score HSF sites within Alus drive antisense transcription was confirmed by ChIP-PCR using ChIP DNA made against HSF1. In response to heat shock, we observed enriched binding of HSF1 at its predicted site compared to the untreated state. The absence of a band in the 'no antibody' (No Ab) control points towards conditional and specific binding of HSF1 to the putative site.

**Figure 9:**
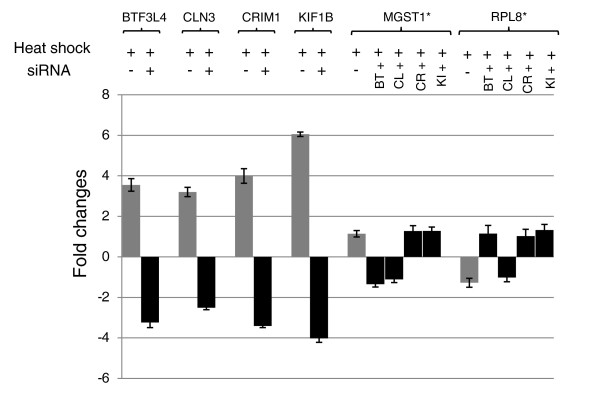
**Quantitative RT-PCR analyses of antisense transcripts after siRNA-mediated knockdown of antisense transcripts**. Elevated levels of antisense transcripts seen in the Affymetrix antisense exon array analysis were confirmed by qRT-PCR for four antisense transcripts. These transcripts showed down-regulation after knockdown of antisense transcripts by specific siRNAs following transient transfection in a cell line. The levels of two control genes (marked by asterisks) unaffected by heat shock and devoid of Alus harboring HSF sites were monitored as experimental controls. These control genes, *MGST1 *and *RPL8*, did not show significant expression differences either during heat shock stress or after siRNA-mediated knockdown of the respective antisense transcripts. Error bars represent standard deviation among experimental replicates.

**Figure 10:**
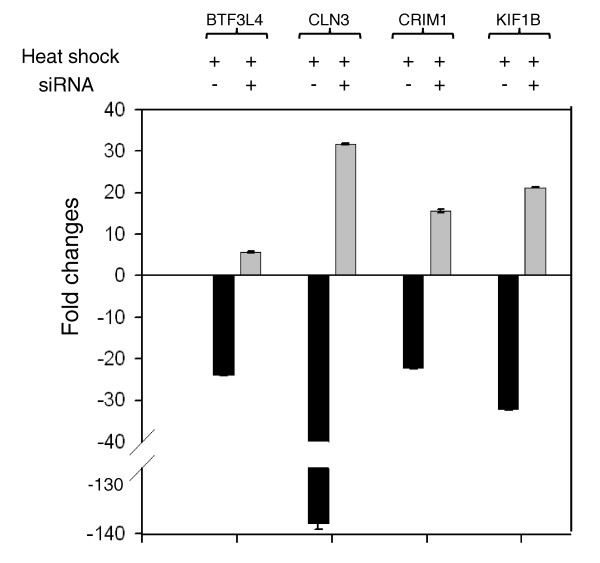
**Quantitative RT-PCR analyses of sense transcripts following siRNA-mediated knockdown of antisense transcripts**. In response to heat shock, the sense transcripts are down-regulated. These transcripts contain an antisense Alu in the genic region with a predicted HSF site that may drive antisense transcription. Following siRNA-mediated knockdown of the antisense transcripts (signal in Affymetrix antisense Exon array), sense transcripts were up-regulated in response to heat shock. Error bars represent standard deviation among experimental replicates.

The apparent down-regulation of sense transcripts during heat shock stress may be modulated by the levels of antisense transcripts being transcribed by nearby Alus harboring HSF sites. To validate our hypothesis, we selected a few down-regulated transcripts that harbored HSF sites in proximal Alus in an antisense orientation. The HSF occupancy of these putative high score HSF sites within Alus after heat shock was confirmed by ChIP-PCR. The enrichment of HSF1 in treated ChIP DNA compared to untreated and 'no antibody' control confirms transient binding of HSF1 to these sites in response to heat shock (Figure 8). The observed faint band in untreated ChIP DNA and treated 'no antibody' control for *CRIM1 *probably indicates antisense transcription of the gene in the normal state also, which is increased during heat shock stress. Subsequently, the differential expression level of antisense transcripts was confirmed by quantitative RT-PCR (qRT-PCR). In response to heat shock we found increased levels of antisense transcripts, which were alleviated after small interfering RNA (siRNA)-mediated knockdown of the antisense transcripts (Figure 9). The levels of two genes, *MGST1 *and *RPL8*, lacking HSF-harboring Alu repeats and unaffected by heat shock were monitored as experimental controls. Prior to siRNA treatment, the down-regulated levels of sense transcripts detected by exon arrays in response to heat shock stress were confirmed by qRT-PCR. siRNA-mediated knockdown of the antisense transcripts led to the up-regulation of expression levels of sense transcripts in response to heat shock stress (Figure 10). The fold-changes observed for antisense transcripts were rather lower compared to sense transcripts. This could possibly be attributed to high transcription turnover of small-sized antisense transcripts compared to full-length sense transcripts. Additionally, reporter constructs with HSF-harboring Alus show, on average, two-fold induction in response to heat shock. This substantiates the expression level changes seen for antisense transcripts.

## Discussion

This study demonstrates a non-random presence of Alu repeats harboring HSF sites in heat shock responsive transcripts and adds a new dimension to the involvement of these elements in transcriptional response to heat shock stress, especially in down-regulation through an antisense-mediated mechanism. In response to heat shock and other stress, for instance viral infection, elevated levels of RNA pol III transcribed SINE non-coding RNAs (ncRNAs), such as Alu RNA and B1 and B2 RNAs [26,32], have been reported. These RNA levels are also coincident with the expression of heat shock protein genes [33]. Further, Alu RNA and B2 RNA have been shown to act as transcriptional co-repressors in response to heat shock through their direct interaction with RNA pol II [27,34]. These results hint at the involvement of Alu in maintaining physiological homeostasis in stress conditions. Our results further demonstrate that Alus harbor functional HSF binding sites, which could further add to the heat shock response regulatory repertoire of these elements.

There is increasing evidence of involvement of Alu elements at various levels of transcription [35,36]. These elements have been shown to harbor a large number of TFBSs as well as hormone responsive elements that have not only been demonstrated to be functionally active but are also enriched in the promoter proximal regions of specific biological processes [3,16,37-40]. Since these elements are also retro-transpositionally active and can be distributed in the genome, in some cases, they also serve to distribute these regulatory sites in the genome and create novel regulatory networks [41-46]. Additionally, Alu elements are also exonized in different transcript isoforms and comprise a bulk of antisense transcripts and the edited transcriptome [47-50]. Besides providing enhancer binding sites for the RNA pol II machinery, Alus are also transcribed through RNA pol III machinery. In light of increasing reports of ncRNA functions in the genome [51-54], these elements, comprising nearly 11% of the genome, cannot be overlooked [20,55]. In our study we observe that Alus containing HSF sites are significantly enriched in the upstream and genic regions of heat shock responsive transcripts. Additionally, like other elements, these also show positional conservation across different subfamilies and most of the sites are present in the right arm monomer of the Alu repeat. Contrasting distributions of HSF sites in the upstream and genic regions are observed. HSF sites in the upstream regions are mostly in non-Alu sequences whereas in genic regions they are mostly enriched in the Alu elements. Most importantly, we observe that even though the relative proportions of Alus in sense and antisense orientations between up- and down-regulated transcripts are relatively constant, the HSF sites are more abundant in Alus that are present in the antisense orientation. This is most striking in the genic regions of the down-regulated transcripts.

**Figure 11:**
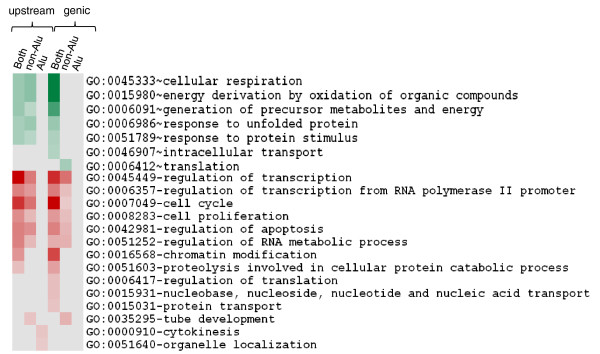
**Gene Ontology analyses of differentially expressed transcripts containing HSF sites in Alu and non-Alu regions**. Heat map of the significantly enriched GO processes (*P*-value cutoff <0.05, after correction) in differentially expressed transcripts. The categories are in relation to Table 2: 'Both', Alu + Non-Alu; 'Non-Alu', exclusively in non-Alus; 'Alu', exclusively in Alus. Green and red colors denote GO processes for up-regulated and down-regulated transcripts, respectively.

Gene Ontology (GO) analysis of the differentially expressed transcripts harboring HSF binding sites, irrespective of their presence in Alu or non-Alu regions, show significant enrichment of processes that are anticipated to vary in response to heat shock stress (Figure 11; Additional file 6). For instance, processes related to unfolded protein response, cellular respiration, and intracellular transport were observed to be up-regulated whereas those related to cell cycle, regulation of transcription, RNA and DNA synthesis processes, regulation of apoptosis, and so on were observed to be down-regulated. Interestingly, for both up-regulated and down-regulated transcripts, similar processes in each category were enriched for both transcripts with HSF sites in upstream regions and those with HSF sites in genic regions. Comparing the distribution of these sites in Alu and non-Alu regions revealed some interesting features. In the up-regulated transcripts, genes with HSF sites in the Alu repeat do not seem to contribute to additional enrichment in specific biological processes. In contrast, in the down-regulated transcripts, when we compare the enrichment of specific biological processes they seem to be more significant when genes with HSF sites from Alu elements are included. This is especially apparent in processes related to regulation of transcription, cell cycle, cell proliferation, and so on. Some of the GO processes show enrichment only when Alus containing HSF sites are considered - for instance, in processes such as chromatin modification, nucleic acid and protein transport (Figure 11). In support of this finding, it is already indicated in the literature that Alus modulate genome-wide chromatin remodeling in response to heat shock [33,56]. However, whether heat shock is a cause or effect is still not clear. Our observations suggest that this could be mediated through HSF binding in Alu repeats. Additionally, we observed 71 up-regulated transcripts that harbored HSF sites exclusively in Alu repeats (Additional file 7). Though most of the transcripts could not be annotated, GO analysis shows that processes for cellular response to stress, ATP biosynthesis and chromatin organization could be modulated by HSF sites in Alu repeats (Table 5).

**Table 5:** GO classification of up-regulated transcripts with HSF sites present exclusively in Alu repeats in the upstream regions

Category	HSF site only in Alu
GO:0033554: cellular response to stress	*SPDYA*, *ATP7A*, *SUMO1P3*, *SFPQ*, *FBXO6*, *EIF2B3*, *FANCB*
GO:0006754: ATP biosynthetic process	*ATP7A*, *ATP1B3*, *ATP11C*
GO:0006325: chromatin organization	*HIST1H2BC*, *HIST1H2BE*, *BPTF*, *SMARCAL1*, *HAT1*, *HIST1H3F*
GO:0044265: cellular macromolecule catabolic process	*PSMD12*, *ISG15*, *SUMO1P3*, *RNASET2*, *FBXO6*, *USP49*, *CNOT4*

Alu repeats also comprise a large fraction of the natural antisense transcripts [50,57-59]. Alus in an antisense orientation with respect to the host gene could be transcribed as RNA (from its own pol III promoter), acting *cis*-antisense to the host gene transcript. Additionally such antisense-oriented Alus could provide HSF binding sites that can act as RNA pol II signals for initiating antisense transcription. We observed that a significant fraction of the down-regulated transcripts (from Illumina array analysis) have antisense transcripts using Affymetrix Exon arrays (Figure 7). Detection of antisense transcripts does give credence to the antisense-mediated regulation of heat shock responsive transcripts. Interestingly, approximately 50% of the detected antisense transcripts have an Alu-harbored HSF site within 5 kb upstream or downstream of the detected signal (Figure 6). This suggests that HSF sites within Alus could be important for response to heat shock stress by initiating transcription of *cis-*antisense transcripts, which might actively down-regulate transcription of sense transcripts. Of the many possible mechanisms that could result in sense/antisense regulation, the most plausible models are transcriptional interference and double-stranded RNA-dependent mechanisms such as RNA interference. Transcriptional interference at antisense loci is well documented in the literature [60], whereas RNA interference initiated by short double-stranded complementary binding between two RNA molecules may be facilitated by the presence of Alu repeats in opposite orientations.

An interesting observation was the specific enrichment of transcripts that are localized to nucleoplasm and are part of TFIIIC and the histone acetyltransferase complex amongst the detected antisense transcripts (Table 4). The B-box of Alu binds the transcription complex TFIIIC, which can be fractionated into two components, TFIIIC1 and TFIIIC2. The cooperative interaction between the two enhances the binding of TFIIIC2 to the B-box, thus elevating transcription [61,62]. It is plausible that an important component of maintaining cellular homeostasis in response to stress is the down-regulation of ncRNA components, which could further result in reduced transcription and translation. We hypothesize that this might have evolved as a mechanism for additional regulation of the heat shock response in primate genomes. Interestingly, regulation of ncRNA levels may be an important mechanism exploited by intracellular parasites to subvert the host response. Recently, it was shown that there is a significant down-regulation of ncRNAs, including Alu RNA, in human macrophages infected with *Leishmania*. It was shown that proteins secreted by the parasite signal the degradation of TFIIIC, which is required for RNA pol III transcription [63,64]. This mechanism might be sensed as a stress signal by the host, which mounts a homeostatic response leading to successful homing of the pathogen. In another study, the p200 protein of *Ehrlichia*, homologs of which are present in human, have targets within Alu repeats [65]. This potentiates them to modulate the human host machinery for successful infection. In primates, the presence of Alus in promoter regions could contribute different TFBSs involved in the immune response against infection [44,66]. As any infection state is sensed by a cell as a condition of stress, the observations made in this study may be relevant to a diverse array of stresses and infection.

We observed HSF sites in Alus in both the upstream and genic regions of heat shock responsive genes. As evident from Figure 5, the contrasting patterns of HSF site distribution in Alu repeats seems to suggest different roles depending on the genomic context. We propose that Alus in the upstream regions could enhance the effect of heat shock by adding more HSF sites and could also, in primate lineages, engage a new, although small, set of genes in the regulatory network. In genic regions, however, the predominance of antisense-oriented Alus (Table 3) and the presence of antisense signals preferentially in close proximity to the HSF sites (Figure 6) could contribute to antisense-mediated down-regulation initiated by Alus in response to heat shock.

## Conclusions

Alu repeats harbor a large number of TFBSs that are functionally active. Through a genome-wide analysis of one such site we have attempted to demonstrate how it could shape existing transcriptional networks. Since these primate-specific elements also retro-transpose and move throughout the genome by non-homologous recombination, they could also create regulatory networks in response to cellular stress or as a response to changing environmental conditions. Alus are transcribed as Alu RNA through RNA pol III machinery and harbor sites for RNA pol II binding. A possibility of cross-talk between the RNA pol III and pol II machineries is increasingly being reported as evidence for the co-existence of these sites [67-69]. Alu repeats might be key mediators of such cross-talk and this might not only be important in physiological homeostasis but also relevant in pathological conditions.

## Materials and methods

### Cell culture

HeLa cells were obtained from the National Center for Cell Science, Pune, India and maintained in Dulbecco's modified Eagle's medium (DMEM) supplemented with heat-inactivated 10% fetal bovine serum from GIBCO (Life Technologies Corporation, Carlsbad, CA, USA) and 1% antibiotic antimycotic solution from HiMedia (Mumbai, India) at 37°C in a 5% CO_2 _and 95% atmosphere incubator. Heat shock stress was induced by subjecting the cells to heat shock at 45°C for 30 minutes in a water bath. Cells were subsequently transferred to the incubator at 37°C and 5% CO_2 _for 2 hours. Before subjecting the cells to heat shock stress, cells were washed with 1 × phosphate-buffered saline twice and then fresh media was added for proper quantification of the heat shock response.

### RNA purification and reverse transcription

Total RNA was isolated from cultured cells using Trizol reagent (Invitrogen, Life Technologies Corporation, Carlsbad, CA, USA) according to the manufacturer's instructions. Subsequent purification using an RNeasy Kit (Qiagen, Hilden, Germany) was carried out to remove residual aromatic compounds that may hinder microarray expression. Total RNA (1 μg) was converted to cDNA using random primers and High Capacity cDNA Reverse Transcription (Applied Biosystems, Life Technologies Corporation, Carlsbad, CA, USA) as per the manufacturer's instructions.

### Microarray experiments

An Illumina TotalPrep RNA Amplification Kit was used to prepare the samples for hybridization on Illumina arrays. Briefly, first-strand cDNA was synthesized from 500 ng of total RNA, followed by simultaneous second-strand cDNA synthesis and degradation of residual RNA with DNA polymerase and RNase H, respectively. *In vitro *transcription (IVT) of the purified cDNA was carried out by T7 RNA polymerase using biotinylated primers. The concentration of the purified cRNA solution was estimated using NanoDrop and then hybridized to WG-6 v2.0 Human Expression BeadChips (Illumina, San Diego, CA, USA) following the manufacturer's instructions. Experiments were carried out using three biological replicates for each treated and untreated condition.

### Quantitative RT-PCR

qRT-PCRs were performed on a 7900HT ABI platform using 2X SYBR Green master mix (ABI, Life Technologies Corporation, Carlsbad, CA, USA). The relative mRNA expression level analyses were carried out using the 2^-ΔΔ^CT method. Three internal controls, *HPRT1*, *RPS18 *and *RPL13A*, were used for normalization during validation by qRT-PCR. These internal controls were selected from our own microarray dataset, based on the criteria of high expression but minimum differential expression. A list of the primers used is provided in Additional file 8.

### Plasmid construction

We considered three genes, *SPN *(G1), *SPINK6 *(G2) and *HIST1H4A *(G3), that harbored high scoring (8.9) HSF binding sites within Alus in the promoter proximal regions for functional validation. For each of these genes, reporter constructs using a firefly luciferase vector with a minimal promoter pGL4.23 (luc2/minP) vector were made with Alus harboring putative HSF sites as insert. An earlier reported functional HSF site in a non-Alu region of the *HSPA1A *promoter was constructed as a positive control. The templates were amplified by PCR from human genomic DNA followed by gel purification of PCR products using Qiagen columns prior to cloning. The clones were confirmed by sequencing.

G1, G2 and G3 clones were subjected to site-directed mutagenesis using a QuikChange II XL kit (Stratagene, Agilent Technologies, Santa Clara, CA, USA) as per the manufacturer's protocol to incorporate desired base changes in the predicted HSF sites within Alus. In each of the clones, the three most-conserved bases were mutated. Site-directed mutagenesis clones were confirmed by sequencing. Details of primer sequences and mutated bases are provided in Additional file 8.

### Transfections and reporter assays

Transient transfections were performed using Lipofectamine 2000 (Invitrogen) as per the manufacturer's specifications in 12-well tissue culture plates. Prior to transfection, 12-well plates were seeded with 2.5 × 10^5 ^cells to achieve optimum confluency. Transfection efficiency was standardized after co-transfection of pGL4.23 firefly luciferase with GFP vector pCM66. Then, 1 μg of each construct in pGL4.23 was co-transfected with 10 ng of pGL4.75 renilla luciferase (Promega, Madison, USA) as a control. Twenty-four hours after transfection, cells were subjected to heat shock stress, as described above, followed by a 2-hour incubation at 37°C and 5% CO_2_. Cells were lysed after 2 hours and luciferase activity was measured with a dual luciferase reporter assay system (Promega) according to the manufacturer's protocol. Each experiment was performed in triplicate and repeated three times. The luciferase activity was quantified using a Tecan Luminometer (Mannedorf, Switzerland).

### Confirmation of HSF binding in Alu repeats through ChIP

In order to confirm the binding of HSF to its cognate sites, we performed ChIP. Heat shock treated HeLa cells as described above were fixed with 1% formaldehyde at 37°C for 10 minutes. ChIP was performed following the protocol provided by Upstate Biotechnology (Norwalk, Connecticut, USA) with modifications as in the Fast ChIP protocol. Goat polyclonal antibody raised against HSF1 was used to immune-precipitate chromatin, along with untreated cells. Briefly, cells were lysed and sonicated using a Misonix 3000 sonicator (Farmingdale, NY, USA) to achieve DNA sizes ranging from 200 to 700 bp. Lysate was pre-cleared using protein-G sepharose beads. Supernatants were then incubated with anti-HSF1 antibody (Santa Cruz Biotechnology, Santa Cruz, CA, USA) at 4°C overnight. Immuno-complexes were purified using herring sperm DNA saturated protein-G sepharose by incubation at 4°C for 3 hours and washed extensively. Chelex-100 resin was used to extract DNA from immune-precipitated chromatin. The cross-linkage between DNA and protein was reversed by heating at 100°C for 10 minutes and protein removed by proteinase K digestion at 55°C for 30 minutes. The immune-precipitated DNAs were recovered using a PCR Purification Kit (Qiagen). In order to confirm the presence of HSF in heat shocked cells and also to confirm the specificity and efficiency of the antibody used for ChIP, we carried out western blotting. The blots were probed with anti-HSF1 (Santa Cruz; 1:2,000). Specific immune-reactive bands were detected using anti-goat secondary antibody conjugated to alkaline phosphatase and detected with BCIP-NBT (Sigma, St. Louis, MO, USA).

The specific binding of HSF1 to its site was further validated by ChIP-PCR in both the Alu and non-Alu regions. Primers were designed for *HIST1H4A*, *SPINK6 *and *ANKRD33 *genes, which have a HSF site within an Alu in the upstream region. *HSPA1A *was used as a positive control as it has a HSF site in a non-Alu region. Input chromatin for both treatments (heat shock versus no heat shock) was used to ensure that an equal amount of chromatin was used for the reactions. In addition, a control ChIP was also performed 'without antibody' to ascertain the enrichment after addition of HSF1 antibody. As a negative control, primers were designed from a region of the genome that does not contain a HSF site and PCR was done with input chromatin, a ChIP sample and no antibody to confirm the specificity. The proximal region of antisense transcripts harboring high-score HSF sites within Alus was also confirmed for its HSF occupancy in response to heat shock by ChIP-PCR. Primers used for all the PCRs are listed in Additional file 8.

### Antisense transcriptome analysis using exon arrays

We carried out two sets of microarray experiments using Exon arrays to detect antisense transcripts in heat shocked cells. In one of the arrays a standard protocol for expression analysis was performed for detection of all transcripts. In the other, specifically antisense transcripts were profiled where the first cycle cDNA synthesis and the IVT amplification process were omitted and the experiment started directly from the second cycle cDNA synthesis [70]. Each of these experiments is described below.

### Detection of sense transcripts

Sense strand expression profiling was performed according to the recommended protocol of Affymetrix's GeneChip Whole Transcript Sense target labeling assay manual (Santa Clara, CA, USA). We used 1 μg of total RNA as starting material. Ambion's Whole Transcript Expression kit (Life Technologies Corporation, Carlsbad, CA, USA) was used for preparation of cDNA. Prior to cDNA preparation, this kit also carries out ribosomal RNA reduction. Briefly, double-stranded cDNA is synthesized with random hexamers (these specifically prime non-ribosomal poly-A and non-poly-A mRNA), coupled with a T7 promoter sequence. This is used for preparation of cRNA through IVT amplification with T7 RNA polymerase. In the second cycle cDNA synthesis, random primers are used in reverse transcription to convert the cRNA into single-stranded DNA. The single-stranded cDNAs (6 μg) are then fragmented, labeled, and hybridized to the array.

### Detection of antisense transcripts

The sample preparation for profiling antisense transcripts was done as described earlier [70]. Compared with the standard protocol, this protocol skips the first cycle cDNA synthesis and the IVT amplification process, and starts directly from the second cycle cDNA synthesis. Since, the IVT step is omitted here, 60 μg of total RNA was used as a starting material before rRNA reduction. Single-stranded cDNA (14 μg) was then fragmented, labeled, and hybridized to the Exon array. The labeled target DNA fragments are in the reverse orientation of the original mRNAs. Thus, hybridization signals will represent transcripts from the same exonic regions but from the opposite DNA strand.

### siRNA-mediated knockdown of antisense transcripts

Antisense transcripts with proximal HSF sites (within 2 kb) were selected for knockdown using siRNAs. For each transcript, three siRNAs were made for the probe selection regions that have signal in the antisense exon array. Primer sequences are listed in Additional file 8. siRNAs were synthesized from Dharmacon (Thermo Scientific, Waltham, MA, USA) and a final concentration of 100 μm/μl was used for the transfection. For each antisense transcript, a mix of three siRNAs was used for transfection to achieve efficient knockdown. Transfection was done using Lipofectamine 2000 (Invitrogen) in 12-well plates, as described above. Twenty-four hours after transfection, cells were treated with heat shock. Following a 2-hour incubation for heat shock response, RNA was isolated from treated and untreated cells. RNA was used for reverse transcription and subsequent real-time PCR for corresponding sense transcripts along with antisense transcripts and control genes, as per the manufacturer's protocol described above.

### Bio-informatics analyses

A large number of computational approaches for analysis of experimental methods and bioinformatics strategies for functional analysis of HSF sites in the genome, in both repetitive and non-repetitive regions, were used. These were carried out using available as well as in-house-developed algorithms in C++ and Perl languages.

### Identification of differentially expressed transcripts in response to heat shock

Illumina BeadChip expression arrays were analyzed using BeadStudio software. Background subtraction was done and average normalization was applied. For selection of differentially expressed genes, we used the Illumina Custom error model and the recommended cutoff of ±13 diff. score.

### Alu repeat analysis in heat shock responsive transcripts

We retrieved the 5 kb upstream and genic sequences of each of the differentially regulated transcripts using the UCSC Table Browser (RefSeq genes track, hg18) [71]. In order to prevent genomic location ambiguity, we filtered out the sequences from the Table Browser data that referred to chromosome type chr*_random and chr*_hap. The coordinates of Alu elements were obtained using the Repeat Masker track of the Table Browser (hg18). The start and end positions of Alu elements in the sense orientation and those in the antisense orientation to the host transcript were classified separately. A random set of RefSeq transcripts were selected and their upstream 5 kb and genic sequences were also retrieved similarly. Comparison of Alu density in the upstream and genic regions of up-regulated and down-regulated transcripts was carried out using a two tailed *t*-test.

### Analysis of distribution of HSF sites in heat shock responsive transcripts

Using an in-house developed tool, Promotif (implemented in C++), HSF sites were detected based on a position weight matrix generated using the Gibbs Motif Sampler. The program generates a score for every site detected [1]. Analysis of documented HSF binding sites [31] upstream of *HSPA1A *gave a score of 8.9 and hence, for our analysis, we set a threshold of 8.7 to locate the most probable biologically significant regions.

HSF sites detected at and above the threshold score were mapped into Alu and non-Alu regions of the upstream and genic regions. The differentially regulated transcripts were thus categorized into three classes for both upstream and genic regions: (i) HSF sites in both non-Alu and Alu regions, (ii) HSF sites in non-Alu regions; and (iii) HSF sites in Alu regions only. In this analysis also we retained Alu strand specificity with respect to the orientation of the host transcript. HSF sites for a given sequence in the upstream region are represented in counts per kilobase. These counts per kilobase were then averaged for the gene number in each class, for example, upstream region and up-regulated transcripts to give counts per kilobase per gene. A scaling factor was applied for genic stretches, and the density was expressed as counts/10 kb/gene. This was done since genic regions are much larger than the upstream 5 kb regions and this scaling makes the genic HSF site density of comparable magnitude to the upstream HSF site density. Comparison of HSF site density between various classes was carried out using a two tailed *t*-test.

### HSF sites in Alu elements

Local alignment of Alu elements with HSF sites in the consensus subfamily sequence from RepBase (release 15.07) was used to assign the actual position of HSF sites within Alu elements. This was performed using the Matcher program from the EMBOSS suite. Positional conservation was checked separately for Alu elements in the sense and antisense orientations.

For generating a consensus motif of the HSF site within Alu subfamilies the 13-bp stretch was retrieved from the upstream/genic Alu sequences of the differentially expressed transcripts. From these, a position-specific scoring matrix was generated using the WEBLOGO program [72]. Motif generation was done separately for upstream and genic sites. We assumed that the upstream and genic Alu elements may be under differential evolutionary pressure and thereby HSF binding sites might have incurred base pair differences. Hence, we predicted the HSF binding sites separately for both the regions. Subsequently, however, we found the HSF sites to be same in the upstream and genic regions.

### Identification of antisense transcripts in heat shocked cells

The status of antisense transcripts in response to heat shock was inferred from the Exon arrays. The Exon array. cel files were analyzed using the Robust Multi-array Analysis algorithm [73] and quantile normalization implemented in the Expression Console software (Affymetrix Inc.). The probe set level information was summarized for the exons from *ab initio *gene predictions in addition to the 'core' and 'extended' exons. To check the quality of the data, we compared the sense and antisense arrays using the various matrices in Expression Console as previously described [70].

Analysis was carried out on a common set of transcripts that were observed to be down-regulated in both the Illumina microarray and Exon arrays in response to heat shock. In this set we next identified the coordinates of HSF sites within the Alus in the genic region through an informatics approach and looked for *cis*-antisense signal in the antisense arrays. A significant detection (*P*-value) of probe signal was considered to count as an antisense signal. In order to correlate antisense signal to HSF binding, we applied a threshold criteria of a 5 kb distance between the HSF site coordinates and the antisense signal (that is, the Exon array probe coordinate). To further the interpretation, we analyzed cases where the HSF site was upstream of the antisense signal.

### Gene Ontology analysis

GO analysis was performed using DAVID [74]. Briefly, we looked for enriched functional categories using the GO-FAT classification as this gives specificity during GO classification by filtering out the broadest terms in the hierarchy. Also, the Functional Annotation Clustering tool was used to summarize annotation from UniProt, InterPro, and the Kyoto Encyclopedia of Genes and Genomes with GO classification to aid functional interpretation of the gene lists.

In accordance with our strategy to determine HSF site distribution in Alu and non-Alu regions (Table 2), we performed GO analyses separately for each of these gene categories. Comparison of genes with HSF sites only in non-Alu regions with genes having HSF sites in both Alu and non-Alu regions allowed us to determine the effect of Alu-harbored HSF sites in terms of GO functional categories.

Illumina whole genome expression profiling BeadChip and Affymetrix Exon array datasets are publicly available on the Gene Expression Omnibus database with accession codes GSE26776 and GSE27127, respectively.

## Abbreviations

bp: base pair; ChIP: chromatin immunoprecipitation; GO: Gene Ontology; HSF: heat shock factor; IVT: in vitro transcription; ncRNA: non-coding RNA; qRT-PCR: quantitative real-time PCR; RNA pol: RNA polymerase; SINE: short interspersed nuclear element; siRNA: small interfering RNA; TFBS: transcription factor binding site; TFIIIC: transcription factor IIIC complex;

## Authors' contributions

MM conceived, designed, supervised, carried out analysis and wrote the manuscript. RP performed all the molecular biology experiments, carried out analysis of microarray and real-time data and participated in manuscript preparation. AKM performed global data analysis and interpretation, including annotation and GO analysis, and participated in manuscript preparation. VJ assisted in bio-informatics analysis, including mapping HSF sites. All authors read and approved the final manuscript.

## Competing interests

The authors declare that they have no competing interests.

## Supplementary Material

Additional file 1 **Genes differentially expressed in response to heat shock stress**. This file contains a list of genes differentially expressed in response to heat shock stress using Illumina BeadChip, analyzed using Beadstudio software. Click here for file

Additional file 2 **Positional conservation of HSF sites**. This file contains the data for the positional preference of HSF sites within Alus in sense and antisense orientations. Click here for file

Additional file 3 **Biased HSF site distribution for Alu orientation in upstream and genic regions**. This file contains HSF site distribution data for Alu and non-Alu sequences in the upstream and genic regions of differentially regulated genes. Click here for file

Additional file 4 **HSF density for upstream and genic regions**. This file contains per gene HSF site density for both Alu and non-Alu sequences in the upstream and genic regions. Click here for file

Additional file 5 **Correlation of HSF sites to antisense signals**. This file contains the HSF sites positions relative to the antisense signal co-ordinates from the exon array for down-regulated genes. Click here for file

Additional file 6 **Gene Ontology analysis**. This file contains the GO category analysis using DAVID for differentially regulated genes. The genes are binned on the basis of the presence of HSF sites exclusively in Alu regions or exclusively in non-Alu regions or in both regions. Click here for file

Additional file 7 **Genes with HSF sites only in Alu regions**. This file contains a list of all the up-regulated genes where the HSF sites in the upstream region are present exclusively in Alu sequences. Click here for file

Additional file 8 **Primers used for validation**. This file contains all the primers used for experimental validation of the inferences from genome-wide expression profiling and subsequent bio-informatics analysis. Click here for file
